# Simulation-based education: what’s it all about?

**DOI:** 10.1007/s40037-017-0354-0

**Published:** 2017-05-30

**Authors:** Jennifer Cleland

**Affiliations:** Centre for Healthcare Education Research and Innovation, Institute of Education for Medical and Dental Sciences, School of Medicine, Dentistry and Nutrition, University of Aberdeen, Aberdeen, UK

**Keywords:** Simulation, Sociocultural, Qualitative, Surgical

## The story

*‘Which way you ought to go depends on where you want to get to …’* Lewis Carroll, Alice in Wonderland

One day at work, I took a ‘cold call’ from a general surgeon, based in my University’s second campus, about 130 miles away from where I am based. Ken and his colleagues had designed the ‘Highland Surgical Boot Camp’, an intensive, four-day simulation experience using experiential learning and hands-on practice to learn new skills and knowledge in a safe environment, based on the principles of a military Boot Camp. While well-established in the USA [1, 2], Highland Surgical Boot Camp was the first of its kind in the UK, aimed at new surgical trainees, and designed to accelerate learners’ transition from the UK Foundation Program (the first two years of generic training after medical school) into the surgical training pathway. The educational content differed from published accounts of Boot Camps as it included simulation-rich training in non-technical and communication skills, as well as operative (technical) surgical skills. Sessions included a ‘wet lab’ (which focused on learning suturing skills), simulated ward round, letter writing sessions and role play of difficult consultations. ‘Memorable case’ narrative sessions were designed to recreate the coffee-room discussions of the apprenticeship model. Formal social events were incorporated into the program and informal socialisation among learners was encouraged.

Ken and his colleagues worked hard to establish this innovative program, and after a couple of successful Boot Camps, had time to catch their breath and start thinking about evaluating Boot Camp. Ken had been given my name as an ‘expert’ in all things medical education research, and asked if I would be interested in planning this evaluation, and applying for a research grant to do so.

I’d always fancied going on a health and fitness ‘Boot Camp’ so the concept very much appealed to me. The problem was that my ‘expert’ reputation really reflects that I am knowledgeable about a couple of narrow topics within the domain of medical education: selection and widening access to medicine, and medical careers decision-making mostly, but not simulation-based education (SBE) or surgical education. I knew next to nothing about SBE, other than reading conference and journal abstracts in a vain effort to have a broad overview of what’s topical in medical education research. What I knew about surgery comes mostly from having a couple of minor operations and being married to an anaesthetist. However, I was intrigued, and wanted to find out more, so I agreed.

With the view of joining ongoing conversations in the field, we wrote a funding bid which focused on individual learning processes. Specifically, and drawing on the work of Tony Artino and his colleagues [3, 4], our project proposed a theory-driven approach, self-regulation learning-microanalysis (SRL-MAT), to assess how participants generated and used feedback about their learning to optimize their strategic pursuit of personal goals during Boot Camp activities. Our study was prospective, assessing attitudes, skills and knowledge before and after specific components of Boot Camp (the wet lab). We obtained a small research grant to support this work but money was limited so I planned to collect the data myself.

And so the study commenced. Our first task was obtaining ethical approval for research activities. These included: pre-Camp telephone interviews with the participants (to assess motivations for signing up, and paying for Boot Camp, as well as very specific questions to do with self-regulated learning). We had ethics permission to record these for later transcription. Participants also completed questionnaires examining factors such as self-regulation and motivation. We incorporated structured observation and questioning during Boot Camp tasks into the project.

Funding was available for me to attend the whole course, and the Faculty were keen that I observe all sessions, to generate new ideas for research and to give them feedback on the educational approaches they had designed and implemented. They talked freely and enthusiastically about why Boot Camp was important and innovative, and how it fitted with wider surgical training. The participants, who I had spoken to in advance on the telephone, talked to me and between themselves over coffees, during breaks, etc. I was also invited me to all the course social events. These included various evening meals and social events, and an outdoor activity (Water sports on a local river. In Scotland. In winter. I politely declined.).

We obtained data which aligned with, and had face validity compared with other work on self-regulated learning. We planned to supplement this with more data from the next Bootcamp as the number of participants attending that particular Boot Camp was small.

## Surprising outcomes

Yet something was missing. To me, the focus of our study did not encapsulate what I had been observing and speaking about to participants and Faculty in my time before and during Bootcamp. I kept thinking about this study, about our data, about what Faculty and trainees had said to me, about the things I had observed and heard. Also, the story had not ended. Ken would phone at regular intervals to keep me in the loop about the timing of the next Boot Camp, telling me about some of the political factors happening in the background which were relevant to Boot Camp’s success or failure.

Reflecting more and more on what I had seen and heard, and the ongoing conversations about the future of Bootcamp, I started to question what we should be evaluating – was Boot Camp all about individual learning processes or was there more to it? It was slowly dawning on me that my lack of SBE knowledge, and my excitement about having an opportunity to use self-regulation theory and measurement, had led me to look at Boot Camp solely in terms of a means of individual, cognitive and acquisitive learning. Of course, this is an essential focus of SBE research, given simulation is a time-efficient way to accelerate learning and prepare trainees for real-life practice in an era of restricted working hours and increasing emphasis on protecting patients from unnecessary harm [5, 6]. However, is it the only perspective worthy of investigation?

In the following months I examined the interview data afresh, and reread the notes I had taken throughout the Boot Camp. Concurrently, I was editing some chapters sent in by colleagues for inclusion into my book with Steve Durning, ‘*Researching Medical Education*’ [7]. These chapters referred to various theories used in exploring and understanding the influence of people and context on learning, as per Vygotsky’s notion that learning is a socially constructed process [8].

The penny dropped: although designed to accelerate individual learning and skills acquisition, surgical Boot Camp was inherently a social activity, bringing together groups of trainees/residents (the learners) and Faculty, in a residential situation away from the everyday clinical environment. By recognizing this explicitly, we could start to understand two things. First, how the relationships between Faculty, participants and activities during Boot Camp influenced learning, the nature and influence of the hidden curriculum [9]. Second, the influence of the particular cultural context, those of the wider sociocultural, institutional, and historical setting and complexities of surgical training, in which Highland Surgical Boot Camp was situated [10–12].

After communicating this new approach to our funder, who generously continued to support the work, and ensuring necessary ethical amendments, we re-positioned our study. Our design shifted from one of individual learning processes, to instead work from the position that learning at Boot Camp was participative rather than merely acquisitive, that environment, rules, tools, and social relations are important to learning and knowing, and there are different valid perspectives on reality [13]. We collected more data scrutinizing Bootcamp from this new angle. The data highlighted the explicit and hidden curricula of Boot Camp, of enculturation and socialization into surgical training, and a way of participants gaining social capital [14] in relation to both progressing in training and seeing what life was like for consultant surgeons.

I will not reiterate the results of the study here as you can find them in the publication of this work [15]. The paper won the inaugural Copenhagen Academy for Medical Education and Simulation (CAMES) prize for innovation in simulation research at the Association for Medical Education Europe (AMEE) later the same month as it was published. I was then invited to keynote on this work at various national and international fora. I hope that this research will lead to SBE architects acknowledging and addressing the social and cultural aspects of learning when planning similar enterprises across healthcare education.

## Lessons learned

So, what did I learn from this process? Quite a number of things.

1. I was interested in self-regulated learning because I rate some of the researchers working in this area very highly. However, individual learning is not my ‘thing’. It never has been! I hated this sort of stuff when I was an undergraduate psychology student (and didn’t exactly shine in the course assessments if I remember correctly). So, my first learning point was to reflect a bit more on my strengths and weaknesses rather than ‘following fashion’ when generating research ideas.
2. The second point is related. I well remember one of my annual reviews as a new lecturer, about 15 years ago. The review process is taken seriously in my institution, and is a good opportunity to take stock and get feedback from a senior member of staff. My reviewer had a reputation for being blunt – and he was. He accused me of being a ‘research butterfly’, fluttering all over the place, doing lots of things but not building a reputation in any one area. I came away chastised and did take his advice. However, I have to reflect on this feedback at regular intervals to rein in my enthusiasm for new things and keep focused.
3. I am going to contradict the above point now. One of the joys of being a researcher is learning ‘new stuff’. I love carving out time to sit and read, to explore the literature, and find out new things, and new ways of looking at things. This is what excites me and informs my papers. I like having qualitative data, scratching my head, and thinking: What does this all mean? What theory could help me pull this together in a coherent and useful way? However, the activities of daily living and working sometimes seem to conspire against my efforts to protect ‘reading time’ in my diary. It is so important though – if I had not known about those theories, if I had not been reading about them at a critical time, then the penny may not have dropped.

## Moral of the story

The moral of my story is as follows. First, it is important to be open to continually learning about alternative ways of seeing the world. New theories can enable us to develop new insights. Second, it is also important to rely on our strengths and to be nimble in the ways we use theory to interpret data. When I intuitively felt that the qualitative data had a different story to tell, one that was not related to our original research question and theoretical framework, I went with this ‘gut reaction’, relied on my qualitative expertise and continued to analyze the data. Third, while it is difficult to ‘abandon’ original research plans, sometimes it is the right thing to do – but (and this is a very pragmatic point) communicate with your funder in a timely manner, and keep them on side! Last but not least – make sure to protect time for your own learning and professional growth.
